# Profile of whole blood gene expression following immune stimulation in a wild passerine

**DOI:** 10.1186/1471-2164-15-533

**Published:** 2014-06-27

**Authors:** Richard Meitern, Reidar Andreson, Peeter Hõrak

**Affiliations:** Department of Zoology, Institute of Ecology and Earth Sciences, Tartu University, Vanemuise 46, 51014 Tartu, Estonia; Department of Bioinformatics, Institute of Molecular and Cell Biology, University of Tartu, Riia 23, 51010 Tartu, Estonia

## Abstract

**Background:**

Immunoecology aims to explain variation among hosts in the strength and efficacy of immunological defences in natural populations. This requires development of biomarkers of the activation of the immune system so that they can be collected non-lethally and sampled from small amounts of easily obtainable tissue. We used transcriptome profiling in wild greenfinches (*Carduelis chloris*) to detect whole blood transcripts that most profoundly indicate upregulation of antimicrobial defences during acute phase response. The more general aim of this study was to obtain a functional annotation of a substantial portion of the greenfinch transcriptome that would enable to gain access to more specific genomic tools in subsequent studies. The birds received either bacterial lipopolysaccharide or saline injections and RNA-seq transcriptional profiling was performed 12 h after treatment to provide initial functional annotation of the transcriptome and assess whole blood response to immune stimulation.

**Results:**

A total of 66,084 transcripts were obtained from *de novo* Trinty assembly, out of which 23,153 could be functionally annotated. Only 1,911 of these were significantly upregulated or downregulated. The manipulation caused marked upregulation of several transcripts related to immune activation. These included avian-specific antimicrobial agents avidin and gallinacin, but also some more general host response genes, such as serum amyloid A protein, lymphocyte antigen 75 and copper-transporting ATPase 1. However, links with avian immunity for most differentially regulated transcripts remained rather hypothetical, as a large set of differentially expressed transcripts lacked functional annotation.

**Conclusions:**

This appears to be the first large scale transcriptional profiling of immune function in passerine birds. The transcriptomic data obtained suggest novel markers for the assessment of the immunological state of wild passerines. Characterizing the function of those possible novel infection markers would assist future vertebrate genome annotation. The extensive sequence information collected enables to identify possible target and housekeeping genes needed to gain access to more specific genomic tools in future studies.

**Electronic supplementary material:**

The online version of this article (doi:10.1186/1471-2164-15-533) contains supplementary material, which is available to authorized users.

## Background

Parasites and pathogens are recognized as a major evolutionary force, and all living organisms face a continual struggle to fend off immunological insults within their environment reviewed in [1, 2]. A host’s ability to resist infection is therefore often seen as a major determinant of fitness in nature [3]. Yet most of our knowledge about the function and dynamics of immune responses comes from laboratory studies of inbred mice in highly controlled environments with limited exposure to infection. Natural populations, on the other hand, exhibit wide genetic and environmental diversity [4]. Immunoecology links patterns of immune responses and disease susceptibility to individual fitness consequences [5–7], and asks how immune defences have evolved, are used and are optimized in different environments, ecological settings and lineages. Integrating genomic information into immunoecological research enables to see how variation in genetic background can be linked to phenotypic variation, allowing insight into genetic architecture of protective immune phenotypes [4, 8]. However, wild vertebrate species with well-understood ecology typically lack genome sequences [9]. To obtain species-specific nucleotide sequences without prohibitive costs and time required for sequencing of a complete genome, gene expression data can be used [10]. Transcriptome data provides direct insight into the functional part of the genome, enabling one to study the genetic basis of phenotypic variation in species that lack reference sequences [11]. Consequently, sequencing the normalized mRNA pools of various non-model organisms has become increasingly popular amongst researchers ([11–15] and references therein).

While greater discovery of rare transcripts can be made by sequencing normalized mRNA pools, sequencing non-normalized samples enables one to obtain valuable information about changes in gene expression [13]. Nevertheless, experiments looking at large scale transcriptional changes in ecological studies are generally restricted to species for which microarrays could be developed [16, 17]. However, obtaining data of gene expression via sequencing rather than using specific microarray hybridization would not only allow detection of novel transcripts and retrieve species-specific data, but would reduce bias in gene expression profiling from possible cross-species hybridization mismatches [16, 18, 19].

Immunoecological studies generally require non-invasive markers to allow longitudinal sampling from small amount of easily obtainable tissue [4]. Hence characterizing gene expression of blood cells seems to be the choice in this field. So far, the majority of studies that have looked at transcriptional changes following an experimentally induced immune challenge in live animals have focused on a few transcripts that are well known to be associated with an immune response [20–24]. However, few of these studies have highlighted large numbers of genes not specifically involved in immune function [17, 25, 26]. Characterizing the full transcriptional profile following an immune challenge would thus facilitate the design of novel and more accurate primers for genes related to immune system activation.

The study species greenfinch, *Carduelis chloris*, is an extensively studied gregarious seed-eating passerine of the Palearctic region that diverged from zebra finches, the closest species with an assembled genome, ~25 MY ago [27]. Plumage coloration of greenfinches is sexually selected [28] and sensitive to infections [29–31]. Greenfinches tolerate captivity well [32], which facilitates research in ecophysiology, e.g. [33], immune function [34, 35], chronic infections [36, 37], oxidative stress [38], behaviour [39] and personality [40]. Currently there is no greenfinch gene expression data in the NCBI nucleotide database. However, such data are required for selecting appropriate qPCR control and target genes in studies of gene expression. Adding transcriptome data to current information about the physiology and ecology of greenfinches would thus facilitate further immunoecological research on this avian model.

We have compared transcriptome expression in immune-challenged vs sham-injected greenfinches 12 h after injection with bacterial lipopolysaccharides (LPS) to see which genes were expressed in the blood during the acute phase response (APR). LPS is a part of the cell wall of gram-negative bacteria, which are universally present in most environments. A challenge with LPS mimics a functionally relevant natural situation. Injection of LPS initiates APR by mimicking the first stages of a bacterial infection without actually resulting in sustained disease reviewed in [41]. The APR has become an important tool in examining the effects of immune activation on the performance and functionality of other condition-dependent life-history traits reviewed in [42]. It constitutes energetically the most expensive part of an immune response [43], characterized by hyperthermia, the release of endogenous proinflammatory cytokines, the release of glucocorticoids and the presentation of sickness behaviour reviewed in [42]. Specifically, we aimed to (i) obtain a functional annotation of a substantial portion of the greenfinch transcriptome, and (ii) identify transcripts significantly upregulated or downregulated in the blood following an immune challenge.

## Methods

Female wild greenfinches were captured in mist-nets at bird feeders in a garden in the city of Tartu, Estonia (58°22′ N, 26°43′ E) on 7th, 8th, 14th and 15th January 2013. The birds were housed indoors in individual cages (27 × 51 × 55 cm) with sand-covered floors in a single room where they could see their neighbours. The average temperature in the aviary during the experiment was 13.4 ± 1.3°C (average values are given with ± standard deviation). The birds were supplied *ad libitum* with sunflower seeds and tap water, and were exposed to a natural day-length cycle using artificial lighting by luminophore tubes. They were released back to their natural habitat on 14th March 2013. The study was conducted under license from the Estonian Ministry of the Environment (Licence # 1–4.1/11/100, issued on 23rd March 2011), and the experiment was approved by the Committee of Animal Experiments at the Estonian Ministry of Agriculture (decision # 95, issued on 17th January 2012).

**Figure 1:**
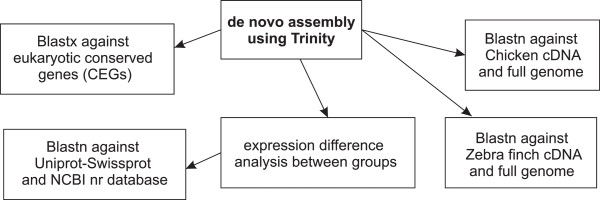
**Summary of the bioinformatics workflow.** After de novo assembly of sequencing data, individual reads were mapped to the obtained assembly i.e. transcript abundance data were calculated per individual. Subsequently expression difference analysis and BLAST searches against Uniprot-Swissprot and NCBI non-redundant database were preformed. BLAST searches were also run against both chicken and zebra finch coding DNA and genome sequences (retrieved from Ensembl).

Prior to the experiment the birds were divided into 2 equal-sized groups on the basis of similar age (yearlings vs. older, determined on the basis of plumage characteristics) and body mass, recorded on 11th March. On the evening of 12th March after the lights had been switched off, four birds received an injection of 0.1 mg E. coli LPS (strain 055:B5, Sigma L2880) in 40 μL sterile isotonic saline into the pectoralis muscle. The dose was based on previous findings of greenfinches where similar treatment affected a number of biochemical health state indices [44]. The remaining four birds received 40 μL isotonic saline injections. Twelve h after injection blood samples were taken and 0.1 ml of whole blood was immediately added to 0.75 ml Tri Reagent BD (Sigma), mixed and stored at −80 C. For the extraction of total RNA, a combination of Tri Reagent BD and Quigen RNeasy Mini kit was used with modifications described elsewhere [45]. The samples were DNase treated according to the instructions of Quigen RNeasy Mini kit. The quality and quantity of the extracted RNA was assessed by Nanodrop and Agilent 2100, respectively. On average 3.8 ± 1.6 μg total RNA with a mean RIN value of 8.8 ± 0.7 was extracted per sample. The total RNA was sent to BaseClear BV (Leiden, Netherlands) for subsequent mRNA extraction, cDNA library construction, sequencing and *de novo* assembly of the filtered reads. Per sample paired-end 51 cycle run was run using a Illumina HiSeq2500 sequencer. Reads in FASTQ format were generated using a Illumina Casava pipeline (version 1.8.3). Initial quality assessment was based on data passing the Illumina Chastity filtering, with ~90% of the reads passing this step. From the remaining reads, ~1% containing adapters (generally considered of low quality) and/or PhiX control signal were removed at Baseclear BV. The second quality assessment was based on the remaining reads (500 million) using the FASTQC quality control tool (version 0.10.0). The average Phred quality score for the reads was 37 ± 0.05, with a read length of 51 bp. The average insert size was 134 ± 10 bp. The sequence reads of individual samples were pooled and assembled using Trinity [46], which is specifically designed for *de novo* assembly of transcriptomes. The assembly has been deposited in the Transcriptome Shotgun Assembly (TSA) database at DDBJ/EMBL/GenBank (accession no. GBCG00000000), the version described here being the first version, GBCG01000000. The following bioinformatics workflow is summarized in Figure 1. The resulting contigs were annotated with Rapsearch2 [47], using the Uniprot-Swissprot and NCBI nr database. Best match was determined comparing bitscores of different alignments. Gene Ontology (GO) terms of the annotated transcripts were obtained and GOSlimViewer [48] was used to generate a high level summary of the GO terms using Generic GO slim Developed by the GO Consortium. The BLAST program [49] (version 2.2.28+) with default parameters was used for BLASTN homology searches against known protein coding DNA (cDNA) and full genome libraries (obtained from Ensembl) of chicken and zebra finch. Coverage of a BLASTN search was calculated by dividing alignment length by transcript length and multiplying by 100. The BLASTX homology search against Core Eukaryotic Genes (CEGs), which consist of 458 conserved genes [50], was used to assess the completeness of the assembled transcriptome. For comparing the data of differentially regulated transcripts with human common host response genes described by Jenner and Young [51], gene name synonyms were obtained from NCBI (http://ftp.ncbi.nlm.nih.gov/gene/DATA/gene_info.gz). All database comparisons used Microsoft Access 2007.

To assess the RNA expression of experimental groups, the reads from each sample were mapped to the assembled transcriptome. Treatment groups were compared by Baggerly's test [52], which calculates the proportion of counts in a group of samples against those of another group, and is suitable for cases where replicates are available in the groups. In the data, a positive fold change indicates upregulation following immune challenge. Expression difference was calculated for all of the contigs. Expression levels presented refer to RPKM (Reads Per Kilobase of transcript per Million mapped reads) separately for both treatment groups.

## Results

*De novo* assembly resulted in 66,084 sequences with a total length of 39.3 million and mean length of 596 bp (N50 = 803, N25 = 1678). The longest and shortest sequences assembled were 13,752 and 201 bp, respectively. Twelve sequences from this dataset were omitted after passing the sequences through NCBI TSA submission contamination screen, on suspicion of bacterial contamination. Around a third of the resulting assembled contigs were successfully annotated using Uniprot-SwissProt (23,151 annotations) with an average identity of 69 ± 21% (the full list of annotated transcripts is given in Additional file 1: Table S1). Only 11,936 of these were unique genes. Setting the e-value to 1E-20 reduced this number to 7,135 (average identity 84 ± 13%, average coverage 43 ± 35%). NCBI nr database enabled to annotate a similar number (24,553) of contigs with an average identity of 81 ± 23%. Altogether, ~44% (28,925) of the assembled contigs found a hit from one or both of the abovementioned databases.

**Figure 2:**
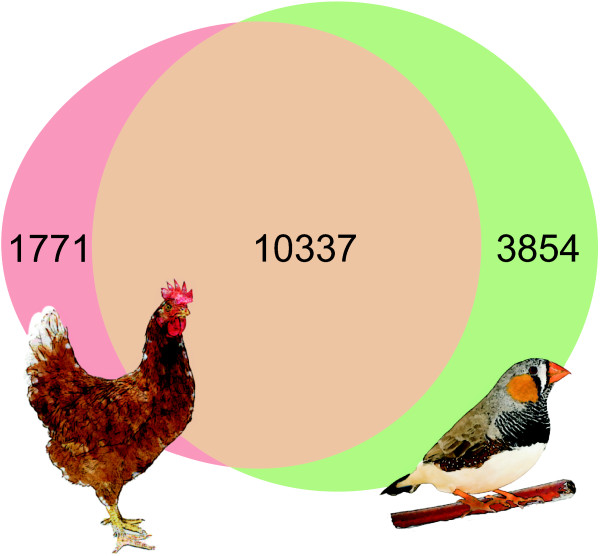
**Comparisons with zebra finch and chicken cDNA.** Comparison of alignment of the 66 072 greenfinch contig sequences with the zebra finch and chicken coding DNA databases (cDNA of known, novel and pseudo gene predictions retrieved from Ensembl). Numbers represent the number of greenfinch contigs aligning to each database. Most of the aligned contigs had a match in both species.

BLASTX results indicated that all of the 458 CEGs were present in our assembled transcriptome, giving an average coverage of 98.3 ± 3.4% at e-value threshold 1E-20. Nevertheless only ~25% of the *de novo* assembled sequences mapped to zebra finch and/or chicken cDNA databases (Figure 2). However, these 25% covered almost half of all the sequences available in the datasets (50% of zebra finch and 42% of chicken cDNAs). The average identities were 96.1 ± 2.8% for zebra finch and 89 ± 4.7% for chicken cDNA sequences. For both species, ~65% of the assemblies had coverage of >80%, indicating good homology. In addition, 86% of the 66,072 contigs mapped to zebra finch genome (70% of those had coverage of > 80%). Strangely, mapping to the chicken genome succeeded only for 27% of the contigs, 24% of which had coverage of >80%. However, changing the BLASTN run parameters (word size reduced to 7, e-value 1E-03) resulted in mapping 65% of the contigs. Only 17% of these had coverage of >80%.

As anticipated, the highest expression was detected for different hemoglobin subunits which made up more than a third from the total unique gene reads per individual. Other highly expressed transcripts (RPKM >1000) included ferritin, histone H5, carbonic anhydrase and RNAs coding various ribosomal proteins, but also included three unannotated transcripts.

**Figure 3:**
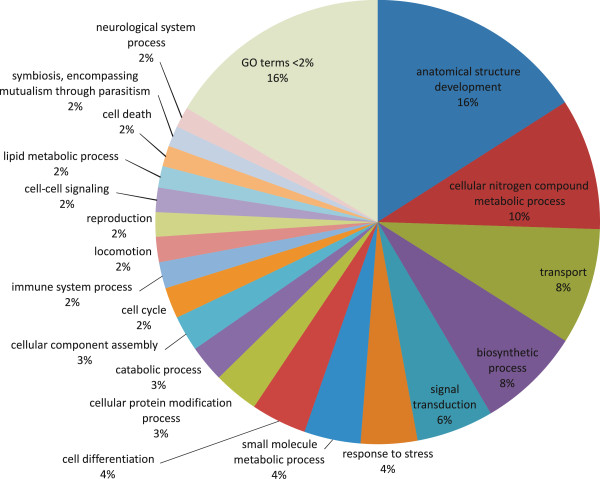
**Gene ontology classification of differentially regulated transcripts.** Biological process gene ontology (GO) terms of the annotated transcripts with absolute fold change >2, Baggerley's test P-value <0.01 (420 genes). This high level summary was obtained using GOSlimViewer [48] with the Generic GO slim set of GO terms developed by the GO Consortium (http://www.geneontology.org). Terms that made up <2% were merged with their parent term. The distribution indicates increase in catabolic processes and biosynthesis.

LPS-injected birds lost significantly more body weight (change in body mass −0.6 ± 0.3 g in LPS injected birds vs 0.4 ± 0.4 g in saline injected birds, t = 3.95, df = 6, p = 0.008), and the experimental procedure significantly affected 1,911 transcripts (absolute fold change >2, Baggerley's test P-value <0.01), of which only 466 (420 unique genes) had been successfully annotated using Uniprot-SwissProt. The summary GO annotation of biological processes of those differentially regulated transcripts is given in Figure 3. Comparing the list of differentially expressed transcripts with 511 human common host response genes showed only 7 shared genes – apolipoprotein B (APOB), DNA polymerase subunit gamma (POLG), interleukin 1 receptor antagonist (IL1RN), cell cycle checkpoint protein (RAD1), zinc finger protein, Y-linked (ZFY), serum amyloid A protein (SAA) and interleukin 8 (IL8). In total, nearly half (247) of the human common host response genes were present in the complete dataset. Chicken cecum transcriptome profile during innate immune response [53, 54] shared upregulation of IL8, SAA, avidin (AVID) and protein MRP-126 (M126) with the current study.

Considering only transcripts with reasonably high expression (RPKM >8, i.e. >1 RPKM per bird), only 54 transcripts were differentially regulated (absolute fold change >2, Baggerley's test P-value <0.001), of which 23 had been previously annotated. Twelve of these annotations were considered sufficiently reliable (identity over 50%, e-value under 1E-20). Fold change and possible function of these 12 transcripts are shown in Table 1. A full list of expression values, test statistics and fold-changes can be accessed from Additional file 1: Table S1.

**Table 1:** **Most significant differentially regulated transcripts**

Uniprot-SwissProt annotation				RPKM			Baggerley's test		
Gene name	Accession	Identity %	Transcript name	LPS ± SD	Saline ± SD	Fold change	Test statistic	P-value	Known or possible functions
GAL2	P46158	91	Gallinacin-2 (Beta-defensin 2)	4.7 ± 2.3	0.02 ± 0.03	221.00	21.4	<0.00001	**antimicrobial activity** [55]
AVID	P02701	70	Avidin	12.3 ± 5	0.2 ± 0.3	53.26	4.8	<0.00001	**antimicrobial activity** [56]
SAA	P02740	73	Serum amyloid A protein	16.1 ± 8.3	0.6 ± 0.3	28.63	3.8	0.00017	chemoattractant for immune cells, induction of pro-inflammatory cytokines and extracellular matrix degrading enzymes [53, 57]
M126	P28318	78	Protein MRP-126	183 ± 76.8	24.1 ± 28.8	7.58	3.9	0.00011	*leukocyte chemoattractant, oxidant scavenging, antimicrobial activity* [58]
DNAJC12	Q9UKB3	78	DnaJ (Hsp40) homolog, subfamily C 12	10.4 ± 5.7	4.5 ± 2.4	2.30	3.7	0.00028	co-chaperone for Hsp70 proteins [59]
HPS5	Q9UPZ3	80	Ruby-eye protein 2	13.5 ± 6.2	6 ± 1.4	2.25	9.1	<0.00001	eumelanin synthesis [60]
LY75	O60449	56	Lymphocyte antigen 75 (C-type lectin, CD205)	62.9 ± 23.2	29.8 ± 12.1	2.14	5.0	<0.00001	antigen uptake of antigen presenting cells [61, 62]
GTF2H1	P32780	90	General transcription factor IIH subunit 1	69.8 ± 32.9	33.8 ± 7.9	2.07	7.5	<0.00001	part of DNA repair complex TFIIH [63]
SLC25A6	P12236	90	ADP/ATP translocase 3 (ANT3)	67.6 ± 15.7	32.9 ± 8	2.06	4.0	0.00008	cellular energy metabolism, mediation of T-cell survival [64]
ATP7A	P70705	90	Copper-transporting ATPase 1	10.6 ± 1.6	5.1 ± 1.3	2.05	7.6	<0.00001	regulation of macrophage function and extracellular superoxide dismutase activity [65]
DSCR3	O14972	88	Down syndrome critical region protein	25.9 ± 2.7	12.9 ± 1.3	2.01	16.3	<0.00001	*part of polymeric immunoglobulin receptor transporter retromere complex* [66]
SLC38A2	Q5F468	89	Sodium-coupled neutral amino acid transporter 2 (SNAT2)	19.3 ± 7.9	49.1 ± 9.5	−2.54	−4.8	<0.00001	cell volume regulation, response to osmotic stress or amino acid depletion [67]

Annotated transcripts significantly up- or down-regulated 12 h after injection of bacterial lipopolysaccharides (LPS). Whole blood mRNA expression of injected female greenfinches was compared with a set of saline injected individuals using Baggerley's test [52]. Only Uniprot-SwissProt annotated transcripts with absolute fold-change >2, RPKM >8, identity >50% and e-values under 1E-20 are shown. Positive fold-change represents higher expression levels in the LPS-injected birds. RPKM values both for LPS and saline injected birds are given with ± SD. Functions related to the immune response and other related processes are reported for the transcripts. If a direct link could not be established, functions from proteins of the same family (according to Uniprot) were attributed to the annotated transcripts (marked in italics). Evidence from avian studies is marked in bold.

## Discussion

This greenfinch gene expression data enabled us to identify possible target and housekeeping genes needed to access to more specific genomic tools in subsequent studies. Although the results incorporate a high number of unreliably annotated sequences, evaluating CEGs from the dataset suggest nearly complete transcriptome coverage. The highly expressed sequences for which no match was found could represent non-coding RNAs that cannot be identified. Even in mammals, a large part of regulatory RNAs is still unidentified [68], so that ~40% of reads map to unannotated regions [16]. Considering also the general bias towards mammalian annotations in public databases, the annotation of only 1/3 of the assembled transcripts is not surprising. Mapping our assembly to zebra finch cDNA database showed similar coverage. Moreover, the majority of transcripts easily mapped to the zebra finch genome, whereas mapping to the chicken genome was rather poor. This discrepancy may reflect differences in phylogenetic distance between the species, as reducing word size improved mapping to the chicken genome. However, problems in short read data assembly are also well known [69]. Therefore reliable annotations could be expected primarily for highly expressed sequences.

Differential expression data showed the transcripts most strongly affected by immune stimulation. To our knowledge, this kind of large scale profiling of immune function has not been done previously on greenfinches or any other passerines. Similarly to immune stimulation of zebrafish (*Danio rerio*) embryos [70], a large proportion (~70%) of differentially expressed sequences lacked a functional annotation, even when only those with higher expression levels were considered. These unannotated transcripts may represent novel immune response genes in birds that need to be checked for this function in subsequent studies.

Although the differentially regulated annotated transcripts with reasonably high expression levels could be tied to immune response or related processes (Table 1), in some cases their exact source and participation in avian immunity remains unclear. This applies especially to the vps26 family protein, DSCR3. Upregulation of mammalian vps26 promotes transcytosis of polymeric immunoglobulin receptor – polymeric immunoglobulin A complex in epithelial cells [66]. However, its upregulation following an *in vivo* immune challenge has not been previously reported. LPS-induced regulation of HPS5 is quite intriguing. HPS5 (Ruby eye-2) is an ubiquitously expressed protein [71] *in vivo* related to melanocyte differentiation and eumelanin synthesis [60]. Its absence influences the distribution of CD63 [72], the platelet activation antigen essential for leukocyte recruitment [73]. Hence, upregulation of HPS5 during the greenfinch immune response suggests the genes involvement in linking melanin-based traits and immune function – a concept proposed in vertebrates [74]. However, the pleiotropic effect of HPS5 in the avian model systems remains to be established.

Upregulation of a conserved transcription initiation factor TFIIH core subunit, GTFIIH1 (p62), as well as the downregulation of SLC38A2 could reflect a global change in cell functioning. Differential expression of SLC38A2 is expected following enhanced proteolysis that causes an increase in transporter substrate amino acids known to inhibit the transcription of SLC38A2 [75] and upregulation of p62 promotes increased transcription by binding to thyroid hormone receptors [76]. Nevertheless, upregulation of the superoxide dismutase activity modulator, ATP7A [65], suggests the need for improved DNA damage repair in response to increased oxidative insult, so that induced expression of the whole TFIIH complex cannot be ruled out (p62 might induce recruitment of other parts of this DNA repair complex [76]). The differential regulation of DNAJC12 may also reflect general transcriptional changes by maintaining the molecular function of estrogen receptors together with HSP70 [77]. Although none of the annotated HSP70 transcripts were upregulated, stress-induced increase in the activity of some HSP70s is achieved by regulating the corresponding HSP40 levels [59].

The upregulation of SLC25A6 could be expected due to its role in promoting Th cell survival [64]. Immunomodulatory role of protein M126 is an expected finding, considering that other members of S100 calgranulin family proteins have known antimicrobial anti-inflammatory roles [58] and the protein is upregulated in chicken cecum after bacterial infection [54]. Similarly, upregulation of GAL2, AVID, SAA and LY75 can be expected, based on some avian immune stimulation studies [53, 61, 78] and our general knowledge about the functions of these proteins reviewed in [55–57, 62]. However the roles of AVID and SAA in avian immunity remain unclear, supposedly together having an anti-inflammatory role by suppressing cell proliferation and supplying host cells with nutrients [53, 79]. Indeed, for several other upregulated transcripts or their protein family members, an anti-inflammatory role has been suggested (M126, i.e. S100 calcgranulins [58], GAL2 i.e. beta-defencins [55], HPS5 [71], DSCR3 i.e. vps26 retromer complex [80]). These findings suggest that 12 h after immunostimulation, anti-inflammatory proteins already dominate at the transcriptomic level. However, while upregulation of M126, GAL2 and LY75 in avian leukocytes is expected, the transcriptional upregulation of SAA and AVID in whole blood suggest that the immune response-related transcriptional upregulation of these common acute-phase proteins in the tissue can be detected. Previously the leukocyte upregulation of SAA following an immune response has been found in human blood [81]. Our results indicate that sauropsid whole blood mRNA sampling also has a diagnostic value in immunoecological studies of small vertebrates, where obtaining sufficient amount of blood for isolating leukocytes is not possible without terminal sampling.

The relative lack of shared genes with human common host response [51] compares favourably with more recent microarray studies involving *in vivo* immune stimulation of gilthead seabream (*Sparus aurata*) skeletal muscle cells, human alveolar macrophages [20] and leukocytes [82]. These studies show regulation of some INF, IL, tumour necrosis factor (TNF) and TLRs, but share only a few upregulated transcripts with our data. Similarly, comparing the list of 420 differentially expressed transcripts with 63 differentially regulated genes identified in a microarray analysis involving LPS administration of chicken liver, muscle and intestinal tissues [83] indicated 3 shared transcripts – IL8, Gallinacin (GAL) and epidermal growth factor receptor (EGFR), although 36 of them were present in the total dataset. While variations in sampling time and quantification methods contribute to these differences, the presence of some low copy-number host response genes could have been masked by the abundance of hemoglobin and other high copy-number erythrocyte transcripts. Moreover, significant inter-host variation in transcript abundance may have masked upregulation of common host response genes as over half of the human common host response genes were present in our dataset with no significant upregulation. Nevertheless, several differentially regulated transcripts have been linked to the immune response of vertebrates and birds, in particular. This, together with the considerable loss of body mass among LPS-injected birds confirms successful immune system stimulation and immune responsive nature of upregulated transcripts in our experiment.

The excess of erythrocyte transcripts in our data allows us to consider their participation in the immune response, as suggested from some *in vitro* studies [23, 84]. In most vertebrate species the principal component of blood, erythrocytes, are nucleated, expressing proteins and mRNAs related to various physiological processes other than oxygen transport [85]. Although it has been long known that non-nucleated erythrocytes participate in an immune response [86], the issue is not well studied [85]. Only recently it has been suggested that chicken red blood cells upregulate toll-like receptor 3 (TLR3), type I interferon's (IFN) and IL8 transcripts in response to viral dsRNA mimetic poly I:C [23, 84]. In rainbow trout (*Oncorhynchus mykiss*) erythrocytes, both heat stress and *in vitro* incubation with LPS modulate genes related to stress, immune response, apoptosis and hematopoiesis [85, 87]. Although upregulation of IL8 coincides with induced expression in chicken erythrocytes by poly I:C [23], no common transcripts with *in vitro* LPS stimulated rainbow trout erythrocytes [85] could be detected. Our data thus suggest that the role of nucleated erythrocytes in LPS-induced immune response *in vivo* is small. Comparison with other tissues is necessary to consolidate this conclusion.

Finally, we were unable to determine the extent to which differences in transcriptome expression between LPS- and saline-injected birds can be ascribed to endotoxin-induced changes in leukocyte numbers. LPS injection usually causes transient changes in the concentration of different types of circulating leukocytes [88]. In domestic chicken, the number of circulating heterophils correlates with mRNA expression of different inflammatory cytokines and chemokines at different time intervals subsequent to corticosterone administration [89]. The question as to whether and how differential gene expression relates to the profile the cellular composition of the blood in non-model species needs to be addressed, preferably correlating within-individual changes in gene expression with corresponding changes in circulating leukocyte counts. Another limitation of our study is that the birds were sampled only at one time-point of the APR, which means that we could have missed other differentially expressed transcripts that appear before and after the 12 h time-point of sampling. Such issues can be addressed by multiple time-point measurements in future studies. Preferably along with increased sequencing depth.

## Conclusions

In passerines, detecting upregulation of antimicrobial defences during acute phase response can be achieved by quantifying whole blood mRNA of SAA, AVID, M126 or GAL2. Quantifying these transcripts along with a selection of housekeeping genes, should provide reliable biomarkers to estimate immune system activation from small blood samples, i.e., in situations where non-terminal sampling is required and only small amounts of tissue can be collected. We also provide the first transcriptome sequencing data of greenfinches, facilitating integration of genomic tools into research involving this species.

### Availability of supporting data

The data sets supporting the results of this article are included within the article and its additional file and available in the DDBJ/EMBL/GenBank under the accession GBCG00000000 (http://www.ncbi.nlm.nih.gov/nuccore/GBCG00000000).

## Electronic supplementary material

Additional file 1: Table S1: Full list of annotated transcripts, expression values, test statistics and fold changes. RPKM values for both treatment groups are given with SD. For each assembled contig annotation with both Uniprot-Swissprot and NCBI- non-redundant database is shown if present. Not all contigs had a match in both databases. (XLSX 6 MB)
